# Prolonged Serotonergic Symptoms in a Pediatric Patient: Suspected Interaction Between Prescription Medications and Kava Supplement

**DOI:** 10.7759/cureus.83966

**Published:** 2025-05-12

**Authors:** Tamas R Peredy, Jeremy Lund, Kathryn E Samai

**Affiliations:** 1 Department of Emergency Medicine, Sarasota Memorial Hospital, Sarasota, USA; 2 Department of Emergency Medicine and Toxicology, Florida State University College of Medicine, Sarasota, USA; 3 Department of Pharmacy, Sarasota Memorial Hospital, Sarasota, USA; 4 Department of Pharmacy, University of North Carolina (UNC) Health, Chapel Hill, USA

**Keywords:** cyproheptadine, drug-drug interaction, duloxetine, kava kava, p450 2d6, piper methysticum, serotonin syndrome, venlafaxine

## Abstract

Kava (*Piper methysticum*) is consumed for a variety of medical and cultural purposes. It is reported to have anxiolytic, muscle relaxant, local anesthetic, and sedative properties. The unregulated use of kava has grown more popular in the United States for a variety of indications and often in combination with traditional pharmaceuticals. A review of existing literature revealed no prior reports of adverse effects from concurrent use of kava and serotonergic agents. We present a pediatric patient who developed prolonged serotonin syndrome after daily use of kava while transitioning from duloxetine to venlafaxine.

A 16-year-old female patient presented to the emergency department with complaints of facial twitching, palpitations, increased anxiety, restlessness, and diaphoresis. Her vital signs were remarkable for tachycardia. Physical examination revealed hyperreflexia and involuntary muscle movements. Her home medications included duloxetine, venlafaxine, aripiprazole, and zolpidem. Over the prior month, the patient had begun taking two different kava preparations for her anxiety. Symptoms were refractory to typical escalating doses of cyproheptadine (18 mg within the first 24 hours) and benzodiazepines (6 mg within the first 24 hours), despite the patient being benzodiazepine naïve. The patient required treatment for 72 hours following discontinuation of serotonergic agents.

This case highlights the importance of pharmacovigilance for significant interactions between herbal products and psychotropics. Several kavalactones have demonstrated significant CYP2D6 and monoamine oxidase inhibition, which in this case may have led to higher neuronal cleft serotonin-norepinephrine reuptake inhibitor drug and active metabolite concentrations. Clinicians should advise patients to limit the use of kava supplements while taking certain prescribed serotonergic medications.

## Introduction

Kava (*Piper methysticum*) is consumed both medicinally and culturally, particularly for its calming effect. Traditionally, kava was prepared as a beverage from the plant’s rhizome, consumed during ceremonial rituals in Pacific Island nations to induce relaxation [1].

In the United States, herbal preparations of kava have gained popularity as a natural alternative to anxiolytic prescription medications [1]. Kava is readily available in a variety of formulations sold by dietary supplement stores, coffee shops, designated bars, and online retailers. It has been reported to have anxiolytic, soporific, muscle relaxant, and local anesthetic properties [2,3]. Clinical trials have confirmed its efficacy for short-term relief of anxiety [4].

Adverse effects [5,6] and pharmacokinetic interactions have been described [7]. A literature search revealed no reports of adverse effects from concurrent use of kava and prescribed serotonergic agents. We present a patient who developed prolonged serotonin symptoms meeting criteria for serotonin syndrome after using escalating doses of kava while being treated for anxiety and depression with duloxetine and venlafaxine, both of which belong to the serotonin-norepinephrine reuptake inhibitor (SNRI) class of antidepressants. They treat depression by increasing concentrations of certain monoamines at their postsynaptic clefts in the brain.

## Case presentation

A 16-year-old female patient presented to the emergency department (ED) with complaints of facial twitching, palpitations, anxiety, restlessness, and diaphoresis. Her past medical history was significant for anxiety, depression, and insomnia for which she was prescribed duloxetine 30 mg daily, venlafaxine XR 75 mg daily, aripiprazole 5 mg daily, and zolpidem 5 mg daily. She had recently been initiated on venlafaxine XR 75 mg, while the dose of duloxetine was simultaneously reduced to 30 mg daily (from 60 mg daily) with the intent to titrate off the duloxetine over a one-week period. She was also prescribed ethinyl estradiol 0.03 mg/norethindrone 1.5 mg daily. She was previously diagnosed with a methylene tetrahydrofolate reductase (MTHFR) polymorphism.

Approximately one month before the presentation, the patient had begun taking kava powder in capsule form. One week prior to the ED visit, the patient had switched to a kava tea extract (Micronesian kava powder, Root of Happiness^TM^, Las Vegas, NV), which was reportedly more potent than the kava-containing capsules. The patient reported recent, sporadic use of a cough/cold product containing guaifenesin, dextromethorphan, and phenylephrine up to a day before presentation to the ED. The patient denied other pharmaceuticals, supplements, or street drug use.

The patient first noticed spontaneous twitching of the mentum one week prior, coinciding with her switch to the more potent kava tea extract. The twitching progressed to spasms of her cheeks and forehead three days prior to presentation. Hours prior to ED arrival, spontaneous movements began affecting her extremities bilaterally.

Upon arrival to the ED, vital signs were heart rate 140 bpm, blood pressure 94/71 mmHg, respiratory rate 20 bpm, and an oral temperature of 98.9°F. Initial physical examination was significant for rapid heart tones, diaphoresis, inducible myoclonus, and 3+ hyperreflexia equally in all four extremities. The electrocardiogram showed sinus tachycardia with normal axis and intervals (R 108 bpm, PR 132 ms, QRS 92 ms, and QTc 457 ms) without ectopy. Laboratory parameters were all within normal limits. Creatine phosphokinase was trended, peaking at 33 U/L (reference normal 26-192 U/L).

The patient was diagnosed by bedside providers with serotonin syndrome. The local poison control center was consulted and concurred. In the ED, the patient was given a liter bolus of normal saline followed by an infusion of dextrose 5%/0.45% sodium chloride with 20 mEq of potassium chloride at 100 mL/hour. Cyproheptadine 12 mg was given orally without demonstrable clinical effect. The patient was admitted to the pediatric ward.

Symptoms of tachycardia, spontaneous facial twitching, hyperreflexia, and myoclonus persisted for 72 hours after admission (Table 1).

**Table 1:** Vital signs

Vitals	Presentation	Hospital day 1	Hospital day 2	Hospital day 3	Hospital day 4	Hospital day 5	Discharge
Temperature min/max (°F)	98.9	98/98.6	97.9/98.6	97.5/99.7	97.9/98.3	97.9/99.2	98.4
Heart rate min/max (bpm)	140	103/159	94/179	69/130	76/139	82/117	113
Respiratory rate min/max (pm)	20	16/24	18/28	18/22	18/20	18/22	18
Blood pressure min/max (mmHg)	94/71	111/75; 122/84	108/78; 125/104	104/83; 124/86	99/73; 115/88	99/64; 110/75	95/63
Oxygen saturation min/max (%)	100	98/100	98/100	98/100	99/100	98/100	100
Oxygen delivery	Room air	Room air	Room air	Room air	Room air	Room air	Room air

Escalating doses of cyproheptadine and lorazepam were given to provide symptomatic relief, peaking on hospital day 3 with a total of 24 mg cyproheptadine and 18 mg lorazepam (Table 2).

**Table 2:** Supportive care medications administered PO: per oral; IV: intravenous

Medications	Hospital day 0	Hospital day 1	Hospital day 2	Hospital day 3	Hospital day 4	Hospital day 5	Hospital day 6
Cyproheptadine	12 mg PO at 18:44	4 mg PO at 10:16	4 mg PO at 4:45	2 mg PO at 00:00	2 mg PO at 00:27	2 mg PO at 01:34	2 mg PO at 01:00
	2 mg PO at 23:54	4 mg PO at 20:13	2 mg PO at 14:15	2 mg PO at 02:30	2 mg PO at 02:24	2 mg PO at 12:33	1 mg PO at 09:25
	-	4 mg PO at 23:18	2 mg PO at 17:55	2 mg PO at 06:19	2 mg PO at 06:40	2 mg PO at 16:36	-
	-	-	2 mg PO at 20:50	2 mg PO at 09:01	2 mg PO at 13:06	2 mg PO at 21:00	-
	-	-	-	2 mg PO at 11:57	-	-	-
	-	-	-	4 mg PO at 14:15	-	-	-
	-	-	-	4 mg PO at 16:19	-	-	-
	-	-	-	4 mg PO at 18:22	-	-	-
	-	-	-	2 mg PO at 20:23	-	-	-
Lorazepam	-	0.5 mg IV at 00:29	1 mg IV at 11:18	2 mg PO at 00:30	2 mg PO at 00:27	-	-
	-	0.5 mg IV at 05:54	2 mg IV at 14:15	2 mg PO at 02:31	2 mg PO at 02:24	-	-
	-	1 mg IV at 10:11	1 mg IV at 16:45	2 mg PO at 09:04	2 mg PO at 04:34	-	-
	-	1 mg IV at 13:16	1 mg IV at 18:35	2 mg PO at 11:58	2 mg PO at 06:40	-	-
	-	1 mg IV at 15:09	1 mg IV at 19:27	2 mg PO at 14:35	2 mg PO at 13:06	-	-
	-	1 mg IV at 15:53	2 mg PO at 20:39	2 mg PO at 16:19	2 mg PO at 15:26	-	-
	-	1 mg IV at 18:19	2 mg PO at 22:47	2 mg PO at 18:22	2 mg PO at 18:38	-	-
	-	1 mg IV at 20:15	-	2 mg PO at 20:24	2 mg PO at 20:58	-	-
	-	1 mg IV at 23:18	-	2 mg PO at 22:22	-	-	-

The patient’s symptoms gradually resolved by hospital day 6, and she was discharged on fluoxetine 10 mg daily. Both the patient and her mother were counseled regarding the use of herbal products.

## Discussion

Serotonin syndrome is diagnosed based on a combination of clinical symptoms and a history of serotonergic medication use. There are no definitive objective laboratory markers. Two sets of diagnostic criteria have been developed to aid clinicians in assessing patients with serotonin syndrome: Sternbach’s and Hunter’s criteria. Developed in 1991, Sternbach’s criteria were studied in a cohort of 38 patients. Ten clinical symptoms and three criteria were identified. Applying Sternbach’s criteria, our patient met the diagnostic criteria: myoclonus, hyperreflexia, diaphoresis, and tremor; and recent serotonergic agent (venlafaxine) initiation [8]. The Hunter criteria were studied in a larger cohort of patients, and a sequential binary branching algorithm was used to arrive at the diagnosis [9]. Utilizing the Hunter criteria, our patient displayed inducible myoclonus upon presentation (along with diaphoresis), satisfying the diagnosis.

In our case, the duration of serotonin syndrome symptoms extended beyond the usually reported 24 hours following discontinuation of the offending serotonergic agent(s) [10]. The severity of symptoms required higher than expected doses of cyproheptadine and benzodiazepines. There are no literature reports of adverse effects from concomitant kava and either duloxetine or venlafaxine. Additionally, to our knowledge, no previous reports of serotonin syndrome induced by kava use alone have been published.

Duloxetine and venlafaxine are metabolized by the CYP2D6 enzyme. Kava extract decreases the activity of CYP2D6 by 73% with in vitro incubation and P450 assays [7]. Duloxetine is metabolized to active and inactive metabolites via CYP2D6 (primary) and CYP2A1 (secondary), with a small fraction excreted unchanged in the urine [11]. Venlafaxine is an inactive parent compound requiring metabolism via CYP2D6 to the active moiety o-desvenlafaxine [12]. In vitro studies have demonstrated Kava’s CYP2D6 inhibitory activity; however, in vivo investigations failed to identify clinically significant CYP2D6 inhibition [13,14]. In this case, the patient was being transitioned from one SNRI (duloxetine) to another (venlafaxine) while reportedly taking escalating concentration kava preparations.

Serotonin and norepinephrine are both partly metabolized by monoamine oxidase enzymes. In vitro data support specific kavalactones, such as yangonin, kavain, and curcumin acting as inhibitors of both the monoamine oxidase A and monoamine oxidase-B enzymes (MAO-B) [15]. In vivo animal studies suggest clinically significant inhibition of the MAO-B enzyme activity [16].

We postulate that the kava lactone-induced CYP2D6 inhibition increased duloxetine concentrations, while MAO enzyme inhibition further increased neuronal cleft serotonin and norepinephrine concentrations. The introduction of venlafaxine finally precipitated the prolonged symptomatic hyperserotonergic state.

Limits of this case report include a lack of testing of SNRI drug and metabolite concentrations during hospitalization, a lack of information about kava dosing, pharmacogenomic testing of CYP2D6 activity, and a comprehensive drug screen to exclude other agents. While the hypothesized CYP2D6 and MAO enzyme mechanisms are biologically plausible, they were not quantitatively analyzed in this case. Patients with some MTHFR gene mutations may have reduced serotonin synthesis and lower serotonin stores [17]. However, the relevance of this polymorphism regarding the susceptibility of developing serotonin syndrome is unknown.

## Conclusions

The timing of the patient’s symptoms supports the clinical concern that the interaction between kava and duloxetine primarily caused the prolonged and severe serotonergic symptoms. As seen in this case, the absence of warnings regarding kava-serotonergic drug interactions may pose risks to future patients. This case emphasizes the need for ongoing pharmacovigilance by pharmacists and prescribers to identify potential interactions between herbal therapies and pharmaceuticals. Clinicians should discourage the use of kava supplements while taking duloxetine.
